# Native femoral anteversion should not be used as reference in cementless total hip arthroplasty with a straight, tapered stem: a retrospective clinical study

**DOI:** 10.1186/s12891-016-1255-9

**Published:** 2016-09-20

**Authors:** Michael Worlicek, Markus Weber, Benjamin Craiovan, Michael Wörner, Florian Völlner, Hans R. Springorum, Joachim Grifka, Tobias Renkawitz

**Affiliations:** Departement of Orthopedic Surgery, University of Regensburg, Asklepios Medical Centre, Kaiser-Karl-V-Allee 3, D-93077 Bad Abbach, Germany

**Keywords:** Hip arthroplasty, Stem version, Combined anteversion, Imageless navigation

## Abstract

**Backround:**

Improper femoral and acetabular component positioning can be associated with instability, impingement, component wear and finally patient dissatisfaction in total hip arthroplasty (THA). The concept of “femur first”/“combined anteversion”, incorporates various aspects of performing a functional optimization of the prosthetic stem and cup position of the stem relative to the cup intraoperatively.

In the present study we asked two questions: (1) Do native femoral anteversion and anteversion of the implant correlate? (2) Do anteversion of the final broach and implant anteversion correlate?

**Methods:**

In a secondary analysis of a prospective controlled trial, a subgroup of 55 patients, who underwent computer-assisted, cementless THA with a straight, tapered stem through an anterolateral, minimally invasive (MIS) approach in a lateral decubitus position were examined retrospectivly. Intraoperative fluoroscopy was used to verify a “best-fit” position of the final broach. An image-free navigation system was used for measurement of the native femoral version, version of the final broach and the final implant. Femoral neck resection height was measured in postoperative CT-scans. This investigation was approved by the local Ethics Commission (No.10-121-0263) and is a secondary analysis of a larger project (DRKS00000739, German Clinical Trials Register May-02–2011).

**Results:**

The mean difference between native femoral version and final implant was 1.9° (+/− 9.5), with a range from −20.7° to 21.5° and a Spearman’s correlation coefficient of 0.39 (*p* < 0.003). In contrast, we observed a mean difference between final broach and implant version of −1.9° (+/− 3.5), with a range from −12.7° to 8.7° and a Spearman’s correlation coefficient of 0.89 (*p* < 0.001). In 83.6 % (46/55) final stem version was outside the normal range as defined by Tönnis (15-20°). The mean femoral neck resection height was 7.3 mm (+/− 5.6). There was no correlation between resection height and version of the implant (Spearman’s correlation coefficient 0.14).

**Conclusion:**

Native femoral version significantly differs from the final anteversion of a cementless, straight, tapered stem and therefore is not a reliable reference in cementless THA. Measuring anteversion of the final “fit and fill” broach is a feasible assistance in order to predict final stem anteversion intraoperatively. There is no correlation between femoral neck resection height and version of the implant.

## Backround

Primary total hip arthroplasty (THA) is one of the most performed orthopedic operations worldwide [1]. Correct component positioning is crucial for postoperative function and outcome [2, 3]. Malpositioning is associated with an increased risk of impingement, dislocation, pelvic osteolysis and wear and early revision. However, the intended cup position is still a matter of debate. So far, most orthopedic surgeons rely on intraoperatively visible or palpable anatomic landmarks and aim to position the cup within an intended target area such as Lewinnek’s “safe zone” [3]. This position can be controlled either visually by eye [4], with the help of intraoperative alignment guides [3] or, more recently computer assisted methods [5].

Different authors have proposed starting with the preparation of the femur (“femur first”/combined antversion) and then adjusting the position of the cup in accordance to the femoral rotation. At the same time, the surgeon has little control about the anteversion of the femoral stem in cementless THA, when using a straight, tapered implant. The femoral component follows the flexion and twist of the proximal femoral channel to a so-called “best-fitting” position [6]. In this context, different studies have reported a high variation in postoperative cementless stem anteversion ranging from −19° retroversion up to 52° anteversion [6–8]. In the following study, we asked three questions: (1) Do native femoral anteversion and anteversion of the implant correlate?

(2) Do anteversion of the final broach and implant anteversion correlate?

(3) Do femoral neck resection height and implant anteversion correlate?

## Methods

The current study is a secondary retrospective analysis of a larger project. In this registered, prospective controlled trial (DRKS00000739, German Clinical Trials Register) patients received a THA with the intraoperative use of an imageless navigation device (Hip 6.0 prototype, BrainLAB Navigation System, Feldkirchen, Germany) [9].

**Fig. 1:**
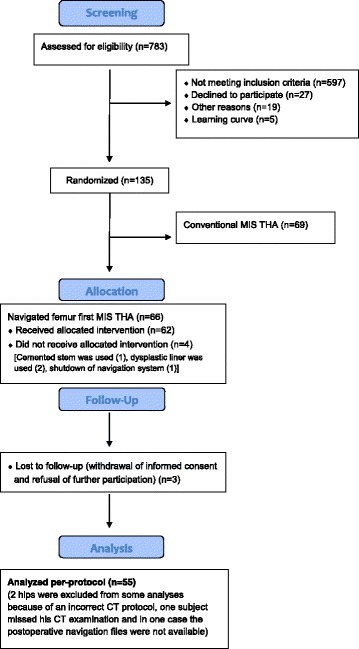
Consolidated Standards for Reporting Trials flow diagram for participants. (MIS, minimally invasive surgery; THA, total hip arthroplasty)

A cohort of 783 patients with osteoarthritis of the hip was screened. The inclusion criteria were: age between 50 and 75 years, an American Society of Anaesthesiologists (ASA) score ≤ 3, unilateral osteoarthritis of the hip (up to Kellgren 2 of the contralateral side), no prior hip surgery, no hip dysplasia or trauma. In total, 597 patients did not meet the inclusion criteria. So in total, a consecutive series of 135 patients were enrolled in this single center study. Out of the 66 navigated THAs 11 had to be excluded (Fig. 1). All in all 55 data sets of navigation-guided group were included for final analysis. Characteristics of the study group are shown in Table 1. After giving written consent, THA was performed by four senior orthopedic surgeons (JG, TR, MW, ES) of the Department of Orthopedic Surgery, Regensburg University Medical Center. All had familiarized themselves with a number of over 200 conventional and navigated THA’s. All operations were performed in the lateral decubitus position through a minimally invasive, modified Smith-Petersen approach [10]. Press-fit cups (Pinnacle, DePuy, Warsaw, Indiana), and cement-free hydroxyapatite-coated stems (Corail; DePuy, Warsaw, Indiana) were used. The Corail stem is a straight, tapered cementless stem that fills the metaphysis and proximal diaphysis in the mediolateral plane. The position of the femoral component is dictated in part by the native femoral neck anteversion, but the final position of the “best-fit” stem is a compromise of fitting a straight stem down the canal of the femur, addressing the flexion and twist of the proximal femur. It is yet unclear, to what extent the anteversion of the final implant can be influenced by the suregeon [6, 8]. The tribological pairing consisted of polyethylene liners and metal heads with a diameter of 32 mm. For the navigation process, reference pins (two Kirschner wires, 3.2 mm diameter) were inserted into the anterior iliac crest and into the ventro-lateral third of the distal femur after stab incisions were made. Dynamic reference bases were then attached to the pins. As a next step, the anterior superior iliac spine (ASIS) and pubic tubercle points were registered using a reference pointer positioned on the skin surface. These points define the reference coordinate system of the pelvis, i.e., anterior pelvic plane and midsagittal plane as the symmetry plane of both ASIS points.

**Table 1:** Characteristics of the study group^a^

	*n* = 55
Gender (female) (%)	32 (58)
Age (yrs)	62,7 (SD 0,6)
BMI (kg/m^2^)	27,1 (SD 4,1)
ASA 1 (%)	8 (14.5)
ASA 2 (%)	32 (58.2)
ASA 3 (%)	15 (27.3)
Treatment side (right) (%)	27 (49)
Femoral componente size (IQR)	12 (2)
Femoral component geometry (%)	Std 27 (48), HO 28 (52)
OP time (min)	71,4 (SD 12,5)

^a^For categorical data values are given as relative and absolute frequencies; for quantitative data values are given as mean with SD in parentheses. *ASA* American Society of Anesthesiologists, *BMI* body mass index, *HO* high-offset stem, *Std* standard stem, *IQR* Interquartile range

**Fig. 2:**
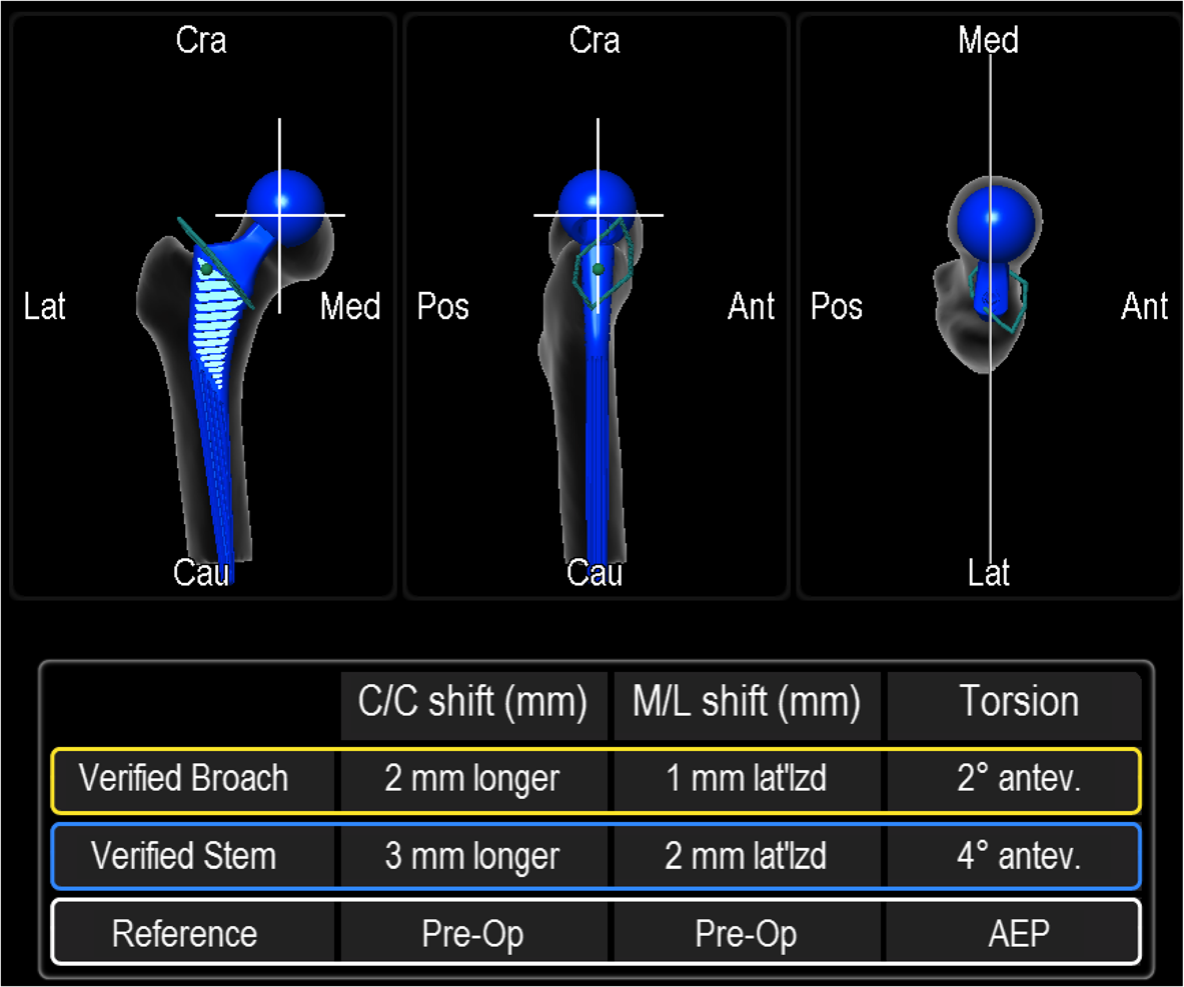
Verified measurements of final broach and implanted stem anteversion with the intraoperative use of an imageless navigation system

**Fig. 3:**
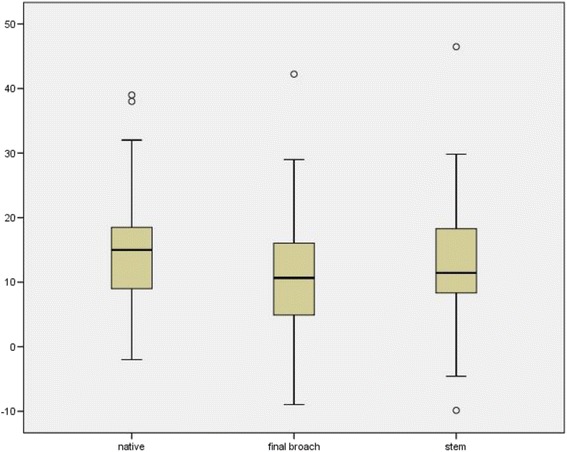
Native femoral version, version of the final broach and stem version in degrees (°) measured intraoperatively with the help of an imageless navigation system (□ = IQR (25–75 %), — = median, ⊥ = 95 % of the results, ○ = outliers)

Native femoral anteversion was measured with the help of the “Ankle-Epicondyles-Piriformis (AEP) plane” as published by Turley et al. [11]. Thereby, the medial and lateral aspect of the epicondyles, the fossa piriformis and ankle points were registered on the femoral side. The knee was flexed 90° during the acquisition of the epicondyles/ankle points. The AEP plane is coincident with the condylar axis and has been shown to be a valid and reliable reference [11]. It is formed by the mid-point of the ankle malleoli, the mid-point of the femoral epicondyles and the piriformis fossa. The normal vector to this plane along with the femoral mechanical axis defines the coronal plane of the femur. The mechanical axis is a line running in the positive direction from the mid-point of the femoral epicondyles to the hip joint center, defining the superior-inferior direction [11]. The native femoral version was measured and saved by the navigation system. After osteotomy of the femoral neck and removal of the head, the femur was exposed. Then the medullary canal was reamed using broaches of ascending size, until one broach reached a stable position. Intraoperative fluoroscopy was used to control the size as well as the cortical “best fit and fill” position of the broach regarding flexion and version of the femur according to two radiographic planes. No attempt was made to achieve a particular rotation. The position of the final broach was measured and saved by the navigation system. Then, the same size, hydroxyapatite coated stem was inserted and the final position was measured and saved by the navigation system (Figs. 2 and 3).

**Fig. 4:**
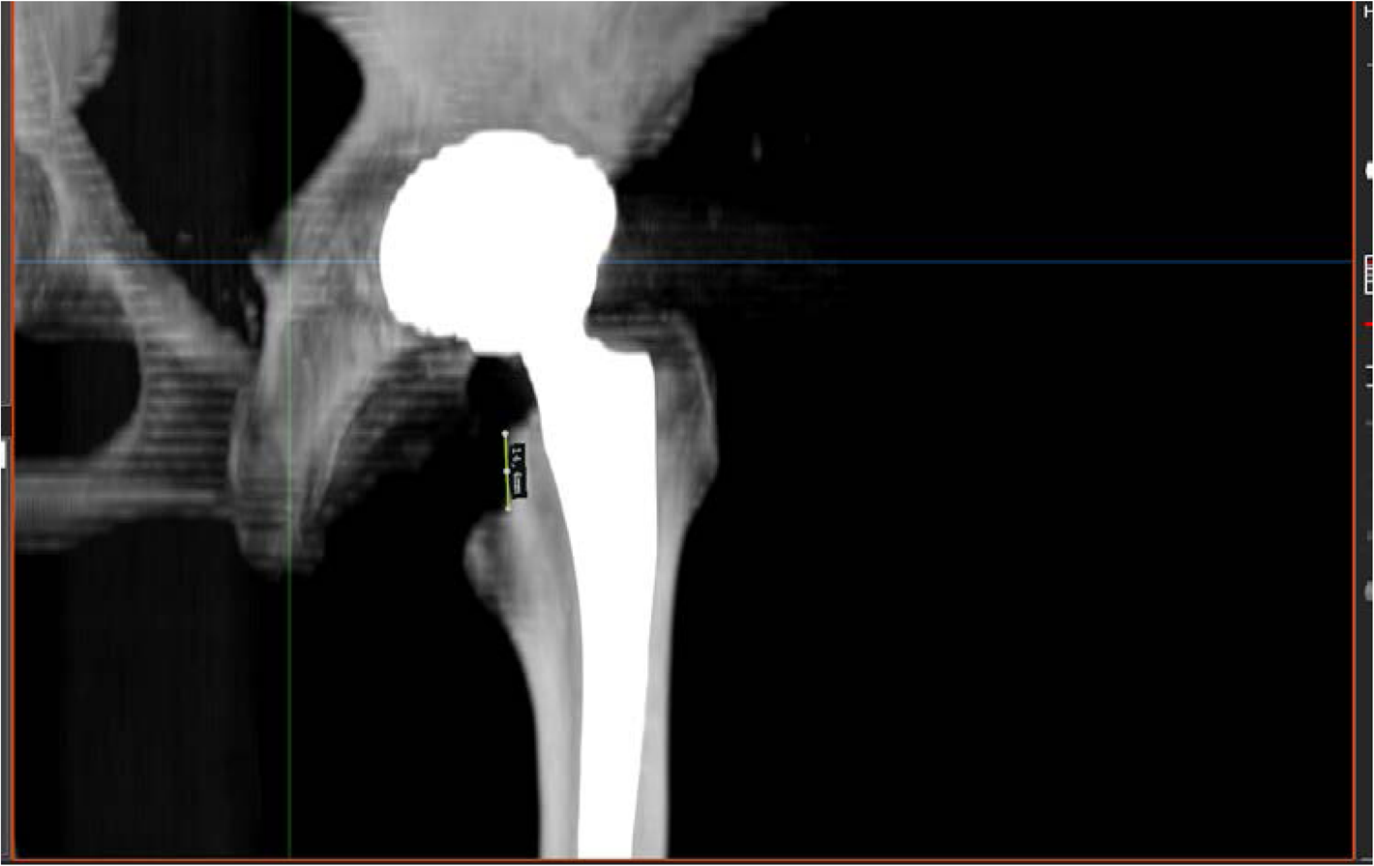
Measurement of the femoral neck resection height by using the ‘semi-automatical’ function of a newly developed digital planning software for CT scans (Modicas, Erlangen, Germany)

The femoral resection height was measured using the ‘semi-automatical’ function of a newly developed digital planning software for CT scans (Modicas, Erlangen, Germany), it was defined as the distance between the deepest point of the resection and the proximal basis of the lesser trochanter (Fig. 4).

### Statistics

Differences between the obtained results of native femoral anteversion, anteversion of the final broach and the implanted femoral component were analyzed descriptively. Means were reported with standard deviations (SD) and 95 % confidence intervals (CI). Correlations were performed using using Spearman’s correlation coefficient due to non-normal distribution of data.

Correlation was characterized as poor (0.00 to 0.20); fair (0.21 to 0.40); moderate (0.42 to 0.60); good (0.62 to 0.80) or excellent (0.81 to 1.00) [12, 13]. Statistical analyses were performed using IBM-SPSS Statistics 22 (SPSS Inc., Chicago, Illinois). A *p*-value <0.05 was considered statistically significant.

## Results

The mean native femoral anteversion was 14.9° (+/− 9.3), with a large range from −2° retroversion to 39° anteversion. The final “fit and fill” broach showed an average anteversion of 11.1° (+/− 9.1) with a range between −9.0° to 46.5°. The mean anteversion of the final femoral stem was 13.0° (+/− 9.5) with a range between −9.9° to 46.5° (Fig. 4). In 83.6 % (46/55), anteversion of the final implant was outside the standard version as defined by Tönnis (15–20°).

**Fig. 5:**
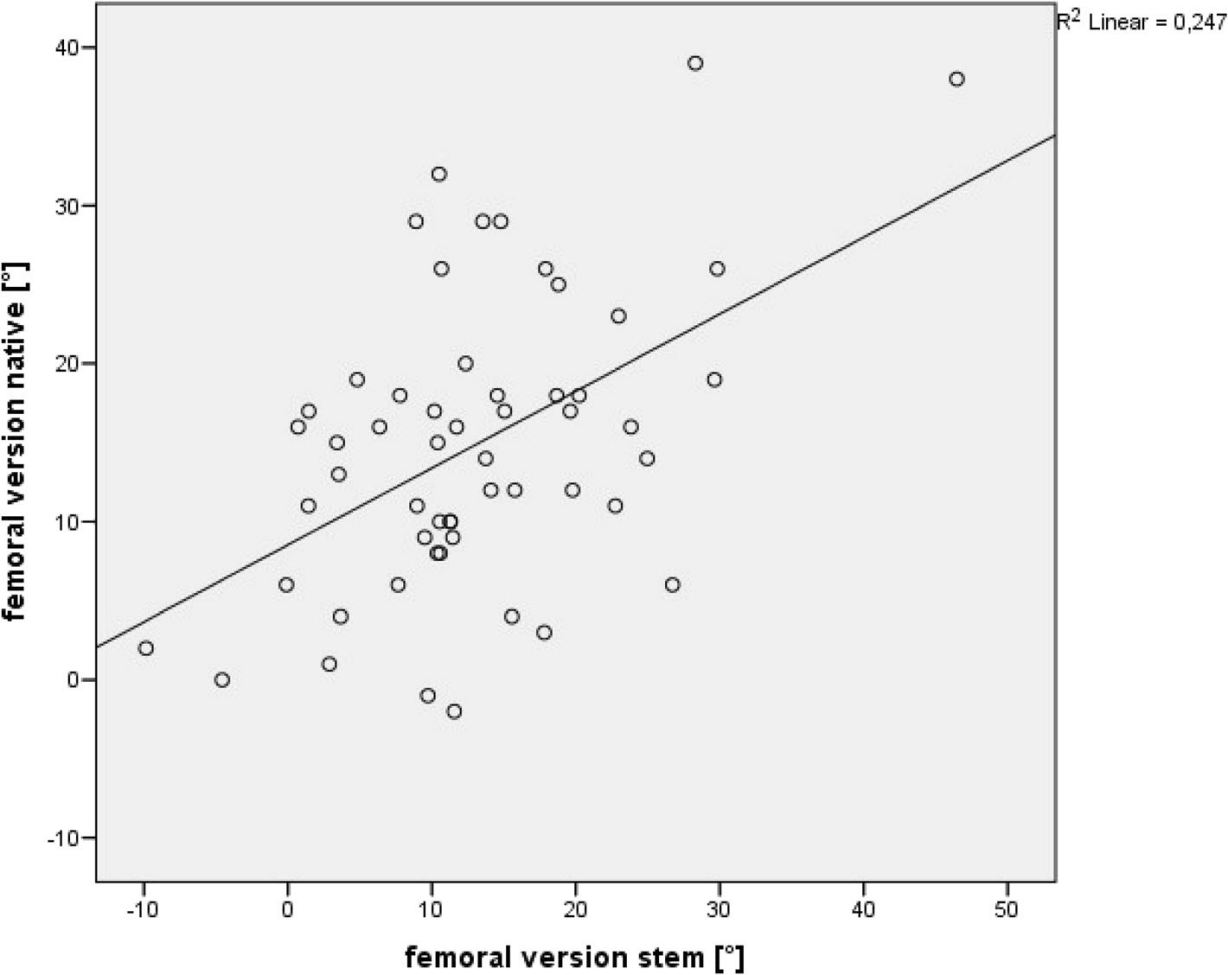
No significant correlation between native femoral version and anteversion of the final implant (Spearman’s correlation coefficient of 0.39 (*p* < 0.003))

**Fig. 6:**
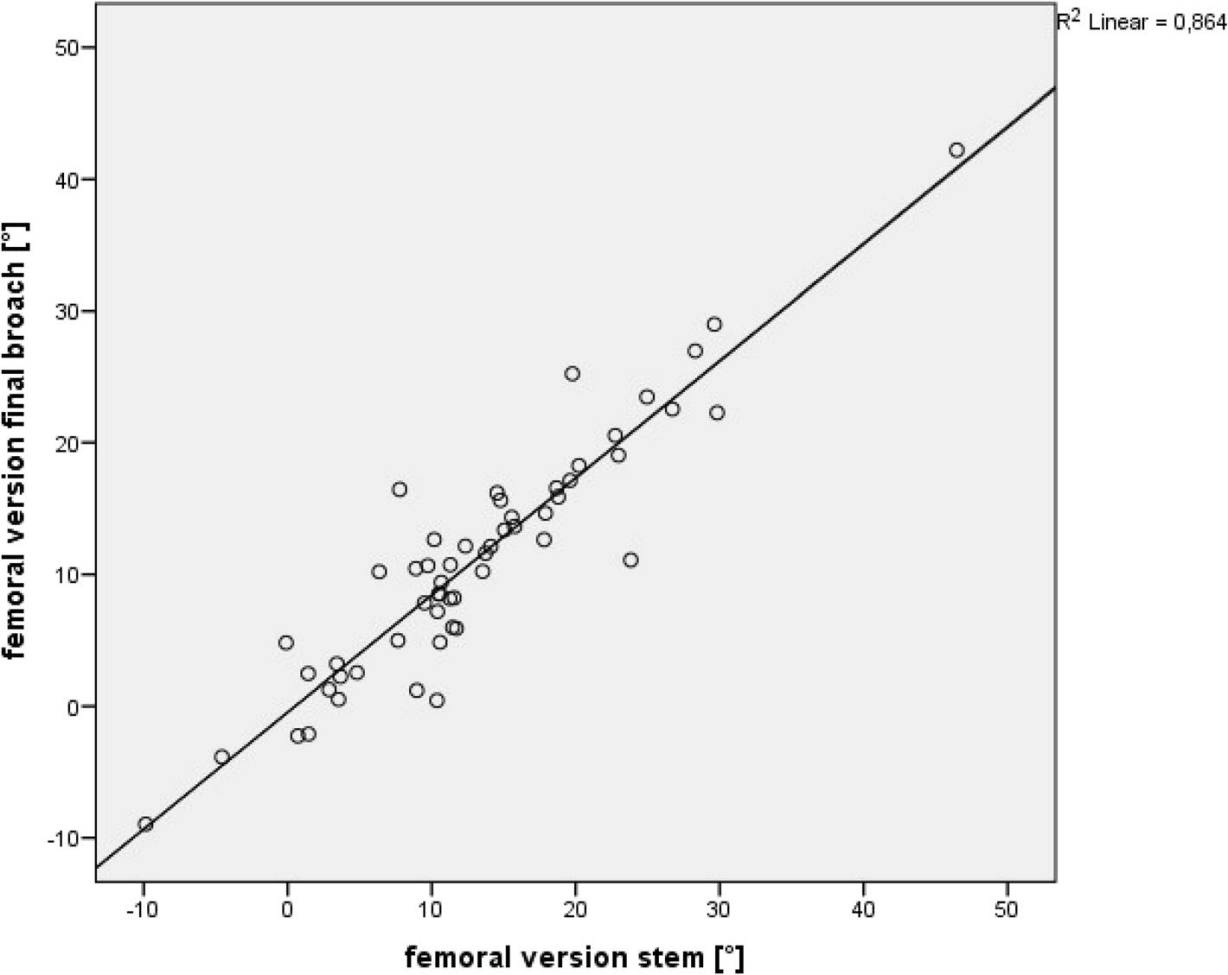
Significant correlation between the anteversion of the final broach and anteversion of a straight hydroxyapatite coated femoral stem was found (Spearman’s correlation coefficient of 0.89 (*p* < 0.001))

The mean difference between the native anteversion and antversion of the final implant was 1.9° (+/− 9.5), with a range from −20.7° to 21.5° and a Spearman’s correlation coefficient of 0.39 (*p* < 0.003) (Fig. 5). In contrast, we observed a mean difference between final broach and implant of −1.9° (+/− 3.5), with a range from -12.7° to 8.7° and a Spearman’s correlation coefficient of 0.89 (*p* < 0.001) (Fig. 6). We found three outliers with a deviation between final broach and final femoral stem of about 10°, which lead to the mentioned range above.

The mean value of the femoral resection height was 7.3 mm (+/− 5.6). There was no correlation between resection height and version of the implant. The Spearman’s correlation coefficient was 0.14.

## Discussion

In answer to the first question posed by this study, we showed that the final position of the stem differs greatly from the native version of the femur up to 21.5°. We found no clinically relevant correlation between the native femoral anteversion and the anteversion of a cementless, hydroxyapatite coated, straight, tapered femoral stem. We therefore conclude, that the orthopedic surgeon should not rely on the native femoral anteversion within the concept of “femur first/combined anteversion” in THA. Even the preoperative knowledge of the native version of the femur would not provide useful information to predict the final prosthetic stem version, at least not for a straight-stem femoral stem as in our study. Consequently, the intraoperative measurement of femoral stem version is crucial for surgeons aiming for an optimized combined anteversion of cup and stem, which directly leads to our second question.

Here, we found a high association with a Spearman’s correlation coefficient of 0.89 (*p* < 0.001) between the rotation of the final broach and the definitive position of the stem. The mean difference between final broach and stem was 1.9° with a standard deviation of 3.5°. In three cases, the difference between the final “fit and fill” broach and the femoral implant was larger then 10°. Analyzing the risk factors for such outliers we found an unusal high femoral anterior/posterior tilt of at least 10° in all of these patients. Since we have shown a direct association between femoral tilt and stem anteversion in the past, these findings confirm that femoral tilt has to be considered for any intraoperative measurements of stem anteversion [14].

As an additional factor which might influence the version of the implant we considered the femoral neck resection height and asked our third question if there is a connection between femoral neck resection height and version of the femoral implant. The correlation between femoral neck resection height and version of the implant was very poor, with a Spearman’s correlation coefficient of 0.14. So there seems to be no relevant impact of femoral neck resection height in matters of the version of the implant.

There are several limitations in our study. First, we used a single cementless stem from one manufacturer in this study. The Corail® stem is a clinically successful implant made of forged titanium alloy (TiAl6V4) [15, 16]. It is a straight implant, with a quadrangular cross section. The proximal part is flared in the sagittal and the coronal plane to provide threedimensional stabilization in the metaphyseal area. Therefore, our findings might not be transferable to other stem designs, like wedge-hip-stems, which provide their stabilization in the diaphyseal area. Second, we used a minimally invasive anterolateral approach with the patient in the lateral decubitus position. Theoretically, the surgical approach (anterior, antero-lateral, lateral or dorsal) might have an impact on the final stem anteversion. Third, the position of the femoral component is dictated in part by the native femoral neck anteversion. So the height of the femoral osteotomy might cause a deviation in femoral component rotation and should be considered in following studies. Fourth, the use of imageless navigation also has general limitations. Particularly in obese patients, pelvic landmarks can become obscured by overlying soft tissue, making direct referencing for computer-assisted surgery difficult [17, 18]. Furthermore, computers are susceptible to crashing, which happened once during our study and are expensive in acquisition and service. Finally, the registration and intraoperative measurement process of navigated THA significantly extends operation time of about 10 min per patient.

Especially in cementless THA a wide range of stem version has been described in literature. Sendtner et al. found a range of the cementless stem from −19° retro- up to 33° anteversion. This is in accordance to the results of Wines et al. and Bargar et al. with a postoperative range of cementles stem version from −15° up to 52° and 1° up to 39° respectively [7, 8]. This is mainly caused by the natural anteroposterior and mediolateral bow of the femoral canal, thickness of the posterior cortex and width of the medullary canal [19–21]. In our study, we were able to confirm this wide range of rotation in cementless stems from −9.9° retro- to 46.5° anteversion. A strength of our study is that we measured native and prosthetic stem version in the same patient position and within the same reference plane. So deviations in recording the data were minimized. Second, we used intraoperative fluoroscopy to verify a “best-fit” position of the final broach in two planes. To our knowledge so far no study has analyzed the trias and association between native femoral anteversion, stem anteversion of the final broach and stem anteversion of a straight, tapered cementless implant. We therefore believe that our trial makes a significant contribution to the understanding of the concept of cementless THA with this stem design and to the idea of “femur first/combined anteversion”.

In conclusion, the native femoral anteversion cannot be used to predict the rotation of the femoral implant, so the surgeon can not rely on a preoperative measurement even by CT-scan. Instead, a “best-fit” final broach from a straight, tapered cementless stem can be used to assess and predict the final stem anteversion for this stem design intraoperatively in order to orientate cup anteversion within the concept of “femur first/combined anteversion” consecutively.

## Abbreviations

ASA: American Society of Anaesthesiologists
ASIS: Anterior superior iliac spine
BMI: Body mass index
CI: Confidence interval
HO: High-offset stem
IQR: Interquartile range
MIS: Minimally invasive
SD: Standard deviaton
Std: Standard stem
THA: Total hip arthroplasty
